# The Influence of Emotional Intelligence on Coping Ability in Senior Female Field-Hockey Players in South Africa

**DOI:** 10.5114/jhk/161550

**Published:** 2023-04-20

**Authors:** Julius Jooste, Ankebé Kruger, Nicola Tinkler

**Affiliations:** 1 Department of Psychology, Northumbria University, Newcastle-upon Tyne, United Kingdom.; 2 Centre for Health and Human Performance, North-West University, Potchefstroom, South Africa.; 3 Physical Activity, Sport, and Recreation Research Focus Area (PhASRec), North-West University, Potchefstroom, South Africa.

**Keywords:** coping ability, emotions, emotional intelligence, psychological demands, sport performance

## Abstract

Research has suggested that coping under pressure could be rooted in the ability to identify and manage one’s emotions. In this study, we investigated this hypothesis using cross-sectional data obtained from a sample of South African national and university level female field-hockey players (N = 60, Mage = 21.57, SD = 3.65). A correlational research design was adopted of which a pen-and-paper survey containing the Emotional Intelligence Scale and Athletic Coping Skills Inventory-28 was used to collect the data. Descriptive results exposed players to yield higher than average levels of total emotional intelligence and coping ability in sport with significant differences noted between the national and university level players in terms of their ability to manage their own emotions (p = 0.018), utilise emotions (p = 0.007, d = 0.74), coping with adversity (p = 0.002, d = 0.84), coachability (p < 0.01, d = 3.17), and overall coping ability (p < 0.01, d = 1.00). After controlling for the level of participation, hierarchical linear regression analyses confirmed the relationship between the study variables exposing total emotional intelligence to be a significant predictor of players’ ability to cope with adversity (β = 0.55, p = 0.006), concentrate (β = 0.43, p = 0.044), maintain confidence and achievement motivation (β = 0.42, p = 0.027), as well as overall coping ability (β = 0.28, p = 0.023). It was concluded that emotional intelligence may be a worthy contributor in the psychological profiling of players and a plausible intervention mapping tool in sport psychology practice to potentially enhance the coping ability of female field-hockey players.

## Introduction

Emotional experiences during sport participation are inevitable with research confirming the illustrious challenges athletes face in their attempt to control variations in mental states that thwart optimal performance (Batinić et al., 2014). The ability to control debilitating emotions in sport forms a crucial part of any athlete’s preparation programme (Soflu et al., 2011) due to the direct impact emotions have on performance outcomes (van Driel and Gantz, 2021). Also, athletes who can cope effectively with their emotions are more capable of competing successfully and achieving optimal levels of performance (Robazza and Ruiz, 2018). As a result, there is a burgeoning interest in the role of emotional intelligence (EI) in sport (Laborde et al., 2016; Maleki et al., 2011; Tinkler et al., 2021, Rubio et al., 2022). Indeed, a ten-year (2008–2018) review of pertinent scholarly work published on the role of EI in sport revealed it to be a valuable quality that benefits athletes and coaches’ performance, the level of psychological skills, and ability to manage aspects that go beyond the sport environment (Magrum et al., 2019).

In this respect, EI broadly refers to a person’s ability to identify and manage one’s own emotions, while also being proficient in distinguishing, understanding, and influencing the emotions of others (Coleman, 2020). Serrat (2017) agrees conceding that a high degree of EI is not only indicative of one’s discernment of the ‘self’ and the emotions of others, but also involves complimentary attributes such as a person’s sense of resilience, optimism, and geniality. Moreover, EI is regarded as a trait-like quality underscored by five distinct features that include self-awareness, self-regulation, motivation, empathy, and social skills (Serrat, 2017). It is conceded that most of these elements are often relied on by athletes in their on-field performance pursuits (Ros et al., 2013) and are fundamental in interactive sport such as field-hockey in which performance is dependent on the team’s collective efforts (Timmerman et al., 2017).

On this point, emotion regulation, stress tolerance, and the ability to overcome adversity are positively coupled with elite-level hockey performance (Kruger, 2010). An athlete who experiences a negative emotion such as frustration due to interruptions in their immediate goals has reduced resources available to cope with the task at hand (Thatcher et al., 2012). One might expect a reduction in coping resources to be more prevalent in less elite or novice athletes, or even perhaps in individual sport athletes as they are likely to exhibit increased anxiety and depression in comparison to team sport athletes (Pluhar et al., 2019). But Nicholls et al. (2012) caution against this line of reasoning as they noted a significant association between emotional intensity and lower levels of coping effectiveness among professional rugby union players. These authors explain that having limited coping resources results in increased use of maladaptive coping strategies (i.e., distancing or disengagement) that have a detrimental effect on athletes’ performance, whether they are experienced, or not (Nicholls et al., 2012). Darvishi and colleagues (2015) support this notion conceding that EI increases stress tolerance and reinforces cognitive capacities for more adaptive coping during competition.

Coping ability is essential in high-level sport participation as it governs athletes’ cognitions and behaviours in response to training and performance demands (Crocker et al., 2015). Coping skills in sport signify athletes’ ability to demonstrate composure, adaptability, and activation control under pressure, which are features that are positively associated with EI (Moradi et al., 2011). Following Pensgaard and Duda’s (2003) initial findings on the connection between coping effectiveness and positive emotions among Olympic athletes, several studies in sport have since verified the predictability of athletes’ maladaptive responses during competition (i.e., poor performance, ill-discipline/transgression of the rules) in the occurrence of a lapse in emotional control (Jones et al., 2005; Robazza and Ruiz, 2018). It is further established that the inability to control emotions may also place athletes at an increased risk of developing injuries, discontent, and potential withdrawal from the sport (Nicholls and Polman, 2007).

It is, therefore, proposed that athletes would benefit from regular assessment of coping strategies and emotions, especially in an altered sport environment that has been marred by added uncertainties and stressors since the onset of the COVID-19 pandemic (Leguizamo et al., 2021). In this respect, research confirms that an elevated level of EI could assist team sport athletes to manage their emotions effectively during high-pressure situations and conflict during competition, while increasing motivation, self-confidence, focus, commitment, and empathy for others (Dalal, 2018; Tinkler et al., 2021). Moreover, when players possess an elevated level of EI, they are believed to be able to stay optimistic in the face of adversity (Laborde et al., 2016), while effectively applying problem-solving skills and enhanced decision-making during games (Abdallat, 2016; Arribas-Galarraga et al., 2020). However, the need arises to further explore and confirm the impact of EI on coping ability in isolated cultural contexts (Laborde et al., 2016) and unexplored sport settings such as field-hockey (Didymus and Fletcher, 2017) to pave the way for supplementary intervention practices that might promote players’ coping in sport and on-field performance. In consideration of extant research on the inherent advantages of EI in sport and the limited literature on female team sport athletes, this study sets out to answer the following research question: is there a significant relationship between EI and coping skill levels in a sample of senior female field-hockey players in South Africa? Based on current literature, it was hypothesized that EI would associate positively and significantly with coping skills and would reveal to have explanatory power in the prediction of players’ coping ability in field-hockey.

Findings from this study may offer worthy impetus for further investigation into the relevance of EI in female field-hockey and the improvement of evidence-based EI interventions to promote coping skills development in sport. Improved coping skills could ultimately contribute to more sustained levels of athletes’ mental health and well-being in sport. Essentially, verification of the relationship between hockey players’ EI and coping ability would appeal to coaches and sport psychology consultants to consider EI as a useful predictor of players’ capability to handle high-pressure sport situations.

## Methods

### Research Design

We adopted a correlational research design and relied on a convenient sampling method to collect the participants’ data using a pen-a-paper survey.

### Participants

An opportunity sample of sixty national/elite (*N* = 26, *M*age = 24.65, *SD* = 3.24) and university/sub-elite (*N* = 34, *M*age = 19.21, *SD* = 1.63) South African female field-hockey players (*M*age = 21.57, *SD* = 3.65) participated in the study. The players’ competitive hockey-playing experience ranged from four to 22 years with player’s positions varying between forwards, midfielders, backs, and goalkeepers. It should be noted that the present study’s sample size is in excess (9.17%) of the minimum sample size recommended (55) by a priori sample size calculation for a hierarchical regression analysis using one predictor variable in block one (level of participation) and one predictor variable in block two (total EIS) with a medium effect size (0.15), 0.05 level of probability, and 80% power (Soper, 2022).

### *Measure Instru*ments

The pen-and-paper survey consisted of demographic items (i.e., age, gender identification, the level of participation, years of field-hockey playing experience), and other standardised psychological measures. The Emotional Intelligence Scale (EIS) (Schutte et al., 1998) and Athletic Coping Skills Inventory-28 (ACSI-28) (Smith et al., 1995) were included in this survey.

**The EIS** is a self-report measure consisting of 33 items that are rated on a 5-point Likert scale ranging from 1 (strongly agree) to 5 (strongly disagree). The EIS is divided into four subscales namely perception of emotion, managing own emotions, managing others’ emotions, and utilization of emotions. Reverse scoring applies for some items (items 5, 28 and 33) with a higher composite score reflecting a higher level of EI (ranging from 33 to 165). Schutte and colleagues (1998) reported excellent internal consistency scores (*r* = 0.87 to 0.90) and acceptable test-retest reliability (*r* = 0.78) for the 33-item scale. The EIS has been widely used in research in sport and has demonstrated satisfactory levels of validity (Lane et al., 2009).

The **ACSI-28** is a multi-faceted psychological skill measure that is used to determine an athlete’s overall coping ability in sport. The scale’s 28 items are measured on a 4-point Likert scale ranging from 0 (almost never) to 3 (almost always) that are equally spread under seven subscales namely coping with adversity, peaking under pressure, goal setting/mental preparation, concentration, freedom from worry, confidence and achievement motivation, as well as coachability. In some cases, reverse scoring applies (questions 3, 7, 10, 12, 19 and 23). Smith and colleagues (1995) have revealed high internal consistencies with alphas of 0.84 (*N* = 594) for males and 0.88 (*N* = 433) for females with excellent test-retest reliability (0.87) after a period of one week in a sample of college athletes (*N* = 94). The ACSI-28 has been effectively utilised for research on athletes in the South African context (Coetzee et al., 2006; Grobbelaar and Eloff, 2011; Jooste et al., 2014).

### Procedures

The present investigation forms part of a larger research study in which the employed measures (EIS; Schutte et al., 1998 and ACSI-28; Smith et al., 1995) were completed in conjunction with other measures in a once-off base-line assessment held at the North-West University (NWU) in Potchefstroom, South Africa. After obtaining ethics approval from the NWU’s Health Research Ethics Committee (HREC) (Ethical number: NWU-00069-18-S1), the national team and university participants along with their team managers and coaches were approached during a training camp in which they were provided a briefing about the study. During the briefing, those present were also provided a study invitation and information sheet that clearly outlined the nature, requirements, benefits, and risk of the study. The players were informed that participation was voluntary and that they were free to withdraw from the study at any point up to formal report on their data. Players were also free to leave out any items in the survey that they did not feel comfortable answering. Written informed consent had to be given to partake in the study. A pen-and-paper survey was completed in a team format during a session that lasted approximately 30 minutes. A member of the research team facilitated this process and was available should there be any questions on the survey items.

### Statistical Analysis

Upon inspection, the dataset showed no missing values. One outlier was spotted using Boxplots (at the interquartile range rule of multiplier 2) and Z-scores (greater than ±3.29), which was winsorised (Tukey, 1979). Then descriptive statistics (minimum, maximum, mean, and standard deviations) were computed for each tested variable using the IBM Statistical Data Processing Package (SPSS) for Windows (Version 28). The Cronbach’s alpha coefficient was computed to determine the reliability of questionnaires/subscales employed in the present study. The employed scales yielded satisfactory to relatively high internal reliability in the current sample with Cronbach’s alpha values ranging from 0.64 to 0.83 except for the ‘utilization of emotions’ subscale of the EIS, which revealed a low internal reliability value (0.58). However, Pallant (2020) recently acknowledged that Cronbach’s alpha values > 0.50 are still adequate for subscales containing less than ten items and should be considered for analyses. The assumptions of normality were evaluated (the data revealed to be normally distributed) whereafter differences between elite and sub-elite players in this study were evaluated using an independent samples *t*-test analyses to guide us on whether to treat the players’ level of participation (national vs. university level) as a confounding variable in the subsequent analyses. Pearson’s product-moment correlation was used to determine the relationship between the variables. Effect sizes from 0.50 to 1.0 were interpreted as large, greater than 0.30 to 0.49 as moderate, and 0.10 to 0.29 as small (Pallant, 2007). To determine the influence of EI on the group’s coping skills, a series of hierarchical linear regression analyses (using the enter method) were conducted with the level of participation added in block one (step 1) and total EI (predictor) in block two (step 2) to assess the influence on each of the coping skill subscales. The necessary assumptions for the respective analysis were met (i.e., linearity between IV and DV, residual values were independent with a constant variance and normal distribution, no influential cases biasing the model). However, to avert infringement of the assumption of multicollinearity between the independent variables, a composite/total EI score was used as the predictor variable instead of its four associated subscales. Considering the small sample size recruited for this study, using a composite EI skill score also allowed for more power in the hierarchical regression model.

## Results

Table 1 presents the descriptive statistics for the study variables and independent sample *t*-test results. In terms of EI, players obtained the highest score in perception of emotion (36.63 ± 4.97) followed by the management of own (36.20 ± 3.42) and others’ emotions (30.75 ± 4.35). Utilization of emotions was the EI subscale with the lowest score (24.50 ± 2.76). The players’ mean total EI score was 128.08 (*SD* = 11.02) out of a maximum of 165, indicating a higher-than-average level of EI (Schutte et al., 2008). In comparison to norm values of athletes’ coping ability scores (Smith et al., 1995), the players’ revealed above-average values for coping with adversity (58.61 ± 19.16), concentration (65.14 ± 18.57), confidence and achievement motivation (70.97 ± 15.98), goal-setting and mental preparation (52.08 ± 22.11), as well as peaking under pressure (69.44 ± 19.20) with a moderate to high total coping ability score of 61.03 (± 11.75). The players’ goal-setting and mental preparation (52.08 ± 22.11) and freedom from worry (45.42 ± 22.26) revealed below-average values. The independent samples *t*-test results (Table 1) identified five significant differences in the tested variables between the elite/national and sub-elite/university players, indicating that national level players were better at managing their own emotions (*M* = 37.38, *SD* = 3.30; *t*(58) = 2.44, *p* = 0.018, *d* = 0.64), whereas university level players were better at utilizing their emotions (*M* = 25.32, *SD* = 2.81; *t*(58) = −2.79, *p* = 0.007, *d* = 0.74), and coping with adversity (*M* = 65.17, *SD* = 16.80; *t*(58) = −3.24, *p* = 0.002, *d* = 0.84). University level players also reported significantly higher coachability (*M* = 82.84, *SD* = 15.88; *t*(51.23)=−12.67, *p* < 0.01, *d* = 3.17) and overall coping skills scores (*M* = 65.58, *SD* = 11.16; *t*(58) = −3.80, *p* < 0.01, *d* = 1.00) compared to the national level players. Based on Cohen’s (1988) conception of effect sizes, the noted differences had either a medium (*d* = 0.5) or large effect (*d* ≥ 0.8). It should be noted that the Levene’s test for equality in variance showed no violations in the distribution of variables for the two groups (*p* > 0.05) except for coachability and perception of emotions. Therefore, equal variance for these two sub-scales was not assumed.

**Table 1:** Descriptive statistics of the study variables and differences between national (elite) and university (sub-elite) level players.

						National Players (N = 26)	University Players (N = 34)	Independent t-test		
Subscales		Min	Max	Mean	SD	Mean (SD)	Mean (SD)	t-value	df	p
Age		17.00	29.00	21.57	3.65	24.65 (3.24)	19.21 (1.63)			
Emotional Intelligence Scale (EIS)	Perception of emotion	24.00	47.00	36.63	4.97	36.77 (6.08)	36.53 (4.02)	0.17	41.06	0.863
	Managing own emotions	28.00	44.00	36.20	3.42	37.38 (3.30)	35.29 (3.27)	2.44	58	0.018^*^
	Managing others’ emotions	17.00	40.00	30.75	4.35	29.65 (4.13)	31.59 (4.39)	−1.74	58	0.088
	Utilization of emotions	19.00	30.00	24.50	2.76	23.42 (2.31)	25.32 (2.81)	−2.79	58	0.007^**^
	Total EIS score	105.00	157.00	128.08	11.02	127.23 (12.07)	128.74 (10.27)	−0.52	58	0.604
Athletic Coping Skills Inventory (ACSI-28)	Coping with adversity	8.33	100.00	58.87	19.16	50.64 (17.78)	65.17 (16.80)	−3.24	58	0.002^**^
	Coachability	25.00	100.00	65.56	23.79	42.95 (8.06)	82.84 (15.88)	−12.67	51.29	0.000^**^
	Concentration	25.00	100.00	65.14	18.57	60.58 (19.94)	68.3 (16.92)	−1.69	58	0.096
	Confidence and achievement motivation	33.33	100.00	70.97	15.98	69.55 (15.62)	72.06 (16.40)	−0.59	58	0.552
	Goal-setting and mental preparation	8.33	100.00	52.08	22.11	47.12 (19.99)	55.88 (23.17)	−1.54	58	0.129
	Peaking under pressure	33.33	100.00	69.44	19.20	67.62 (17.21)	70.83 (20.74	−0.64	58	0.526
	Freedom from worry	0.00	91.66	45.42	22.26	47.12 (22.23)	44.12 (22.52)	0.51	58	0.609
	Total coping skills	35.71	84.52	61.03	11.75	55.08 (9.82)	65.58 (11.16)	−3.80	58	0.000^*^

Min = minimum; Max = maximum; M = mean; SD = standard deviation; * Statistically significant at p < 0.05; ** Statistically significant at p < 0.01

The Pearson's correlation coefficient analyses revealed a significant positive association between the EIS’ perception of emotion and coping with adversity (*r* = 0.33, *p* = 0.010) and concentration (*r* = 0.27, *p* = 0.037) subscales (Table 2). Managing one’s own emotions associated positively with confidence and achievement motivation (*r* = 0.42, *p* < 0.01) and negatively with coachability (*r* = −0.26, *p* = 0.047). Managing others’ emotions associated positively with confidence and achievement motivation (*r* = 0.28, *p* = 0.028), as well as with total coping ability (*r* = 0.27, *p* = 0.035). Additionally, a significant positive association between utilization of emotions and coachability of players (*r* = 0.31, *p* = 0.016) was revealed. Lastly, a positive correlation was observed between players’ total EI and coping with adversity (*r* = 0.35, *p* = 0.006), concentration (*r* = 0.27, *p* = 0.036), confidence and achievement motivation (*r* = 0.29, *p* = 0.023) and total coping ability (*r* = 0.30, *p* = 0.022). However, all the noted significant correlations had an effect size ranging from low to moderate.

**Table 2:** Pearson product-moment correlations between EI and coping skill subscales.

Emotional intelligence subscales	Coping skill subscales							
	Coping with adversity	Peaking under pressure	Goal setting /Mental preparation	Concentration	Freedom from worry	Confidence and achievement motivation	Coachability	Total coping
Perception of emotion	0.33^**^	0.19	−0.00	0.27^*^	−0.00	0.07	0.01	0.20
Managing own emotions	0.20	0.14	0.13	0.14	−0.07	0.42^**^	−0.26^*^	0.13
Managing others’ emotions	0.24	0.09	0.15	0.14	0.05	0.28^*^	0.19	0.27^*^
Utilization of emotions	0.19	0.03	0.19	0.20	−0.10	0.08	0.3*	0.23
Total emotional intelligence	0.35^**^	0.17	0.15	0.27^*^	−0.03	0.29^*^	0.08	0.30^*^

* Correlation is significant at p ≤ 0.05; ** Correlation is significant at p < 0.01

To further explore the relationship between players’ EI and coping skills in sport, a series of hierarchical regression analyses were performed. Based on the differences noted in the study variables between elite and sub-elite players, the level of participation for the total sample was controlled for and entered in block one (Step 1) with total EI (predictor) entered in block two (Step 2) (Table 3). Players’ total EI revealed to be a significant predictor [F(2, 57) = 9.94, *p* < 0.01] of their coping with adversity (*β* = 0.55, *p* = 0.006) and accounted for 10.6% of the variance in this variable. The players’ total EI score also revealed to be a significant predictor of their concentration ability [F(2, 57) = 3.63, *p* = 0.033] (*β* = 0.43, *p* = 0.044) and confidence and achievement motivation [F(2, 57) = 2.78, *p* = 0.027] (*β* = 0.42, *p* = 0.027) explaining 6.6% and 8.3% of the variance in these variables, respectively. Furthermore, total EI emerged to be a significant predictor of the players’ overall coping skills score [F(2, 57) = 10.53, *p* < 0.01] accounting for 7% of the variance in their overall coping ability (*β* = 0.28, *p* = 0.023).

**Table 3:** Summary of hierarchical linear regression analyses for the influence of EI on coping skills after controlling for level of participation.

Dependent Variable	
Coping with Adversity	*∆R^2^ = 0.259, ∆R^2^* change = 0.106, *F*(2, 57) = 9.941 Total EI β = 0.55^*^
Coachability	*∆R^2^ = 0.703, ∆R^2^* change = 0.00, *F*(2, 57) = 67.304 Total EI β = 0.044
Concentration	*∆R^2^ = 0.113, ∆R^2^* change = 0.066, *F*(2, 57) = 3.632 Total EI β = 0.434^*^
Confidence & Achievement Motivation	*∆R^2^ = 0.089, ∆R^2^* change = 0.083, *F*(2, 57) = 2.782 Total EI β = 0.418^*^
Goalsetting & Imagery	*∆R^2^ = 0.057, ∆R^2^* change = 0.018, *F*(2, 57) = 1.730 Total EI β = 0.269
Peaking under Pressure	*∆R^2^ = 0.034, ∆R^2^* change = 0.028, *F*(2, 57) = 1.017 Total EI β = 0.290
Freedom from Worry	*∆R^2^ = 0.005, ∆R^2^* change = 0.001, *F*(2, 57) = 0.147 Total EI β = −0.049
Total Coping Ability	*∆R^2^ = 0.270, ∆R^2^* change = 0.070, *F*(2, 57) = 10.525 Total EI β = 0.283^*^

* *Statistically significant at p < 0.05; β = Beta of standardized coefficients*

## Discussion

This study sought to examine the link between EI and sport coping ability of senior female field-hockey players in South Africa. The results confirmed our hypothesis revealing strong parallels between EI and field-hockey players’ coping ability. More specifically, a moderately positive correlation was noted between the players’ perception of emotion and coping with adversity, as well as their ability to concentrate. Prior research corroborates our findings suggesting that the ability to recognize and correctly express one’s thoughts and feelings could enable athletes to make more accurate decisions during stressful and adverse on-field situations (Fletcher and Sarkar, 2012; Tamminen et al., 2016). It is also suggested that elite athletes are conditioned to perceive demanding situations as a learning phase while recognizing and using their emotions to focus on the constructive components during adversity (Galli and Vealey, 2008). This idea aligns with the positive association noted between the players’ perception of emotion and their ability to concentrate. In this respect, McCarthy and colleagues (2012) are of the view that adequate awareness of one’s thoughts and feelings helps to align attention towards task-relevant cues. These authors also claim that the regulation of emotions may increase the occurrence of positive feelings such as amusement, contentment, and serenity, which can promote athletes’ ability to direct attentional resources when most needed during competition (McCarthy et al., 2012). These emotions are also associated with a broader and relaxed focus of attention (Fredrickson and Branigan, 2005) that permits athletes to make split-second decisions and to have an optimal sensitivity and readiness for action responses during games (Wilson et al., 2006). On the other hand, ambiguous and unpleasant emotions such as anxiety may cause interfering thoughts to divert attention from task-relevant cues and unnecessarily consume one’s available cognitive resources (Correia and Rosado, 2019). Therefore, it is reasonable to concede that field-hockey players who can identify, perceive, and correctly express their emotions, are more likely to cope with adversity and apply concentration strategies during training and competition.

The findings of the present study also revealed a positive association between players’ ability to manage their own emotions and levels of confidence and achievement motivation. This finding echoes the views proffered from a similar investigation that confirmed the utility of learning to manage one’s emotions as a facilitator of athletes’ positive perceptions about their skills and abilities (Rajeshwari and Raj, 2017). Likewise, Serrat (2017) pointed out that self-awareness and self-motivation are subsidiary domains of EI, which acknowledge the athletes’ striving tendency to meet a desired standard of excellence (achievement motivation). From this perspective, the ability to manage one's own emotions allows athletes to potentially replace unconducive emotions with more adaptive and motivational thoughts during performance endeavours (Jones, 2003). That being the case, it is believed that players in the present study demonstrated increased levels of self-confidence and achievement motivation by merely being self-aware and in control of their emotional states, which is essential in obtaining/sustaining optimal performance in high-level sport (Dupee et al., 2016).

Interestingly, participants’ ability to manage their emotions associated negatively with their level of coachability. Coachability is perceived as an athlete’s openness and susceptibility toward the coach’s feedback and critique (Davis, 2021). The primary role of a coach’s feedback is to reinforce appropriate actions, teach new skills, and motivate athletes to optimise their potential (Nash et al., 2017). Contrary to our findings, Favor’s (2011) judgments on college softball athletes indicated that coaches perceived highly coachable players to be more emotionally stable and agreeable than their less coachable teammates. However, the literature suggests that the communication style preferences of females are underscored by feelings over solutions, which imply that emotional support is more important than instructions on how to master a task (Merchant, 2012). For that reason, it appears that players in the present study value how feedback makes them feel as to what is being said, which could influence their openness and reliance on coaches’ feedback in general. The players' inclination to be reliant on coach instruction and feedback may become less as the proficiency in managing their emotions increases based on the belief that coaching at a senior/high-performance level of participation serves more of an emotional-supportive rather than an instructional purpose (Schmidt and Lee, 2019). Even so, it should be noted that players’ utilization of emotions associated positively with their level of coachability, which on the other hand suggests that players who are more skilled at utilizing their emotions could have a more positive interpretation of their coach’s feedback for the sake of their development. This is not surprising as it is expected that players who are more aware of their own emotions are disposed to remain flexible during problem-solving, as well as more open and susceptible to coaches’ feedback and critique to improve. This finding is sustained by other researchers who agree that coaching received at high-level sport also shapes the development of essential critical thinking skills that tend to promote athletes’ self-reflection, problem-solving, and more logical reasoning in sport (Nash et al., 2017).

Furthermore, our findings revealed a significant association between the players’ ability to manage others’ emotions and their own coping ability, confidence and achievement motivation. The ability to manage others’ emotions refers to actions that are aimed at maintaining or improving the moods of other people (Ciarrochi et al., 2000; Mayer and Salovey, 1997). This also implies the ability to understand and share the feelings of another, which is regarded as empathy (Schutte et al., 1998). Empathy is viewed as the ability to recognise, understand, and respond adaptively to others’ emotions that allows for a deeper level of interaction and encourages prosocial behaviour (Spreng et al., 2009). Literature sustains the strong ties between empathy and coping strategies, notably in cases of perceived psychological well-being (Carnicer and Calderón, 2014) and active coping behaviours that include support seeking and problem-solving (Carlo et al., 2012). Therefore, it is possible that within a team setup (e.g., field-hockey), players who can display higher empathy are more likely to cope due to the restorative influence imparted on fellow teammates’ moods, making them more dependable in dealing with arising issues and challenges. Supporting our findings, sport studies also suggest that good social skills and effective communication within a team environment help athletes to establish mutual goals that enhance their motivation (Savardelavar et al., 2017). On this point, positive links between empathy and self-confidence were noted among student-athletes (Dobersek and Arellano, 2017) with Singer (2006) clarifying that the exhibition of empathy promotes interaction that reduces the perception of fear and intimation, and inadvertently increases the likelihood of an emotional bond within a group (Singer, 2006). Thus, it is logical to accept that empathy as a component of managing others’ emotions, could give rise to social behaviour in a group resulting in higher athlete experiences of confidence and achievement motivation. Finally, the significant explanatory power of total EI on players’ dimensions of coping with adversity, concentration, confidence and achievement motivation, and overall coping ability resonates with earlier research demarking the strong relationship between elevated levels of EI and the use of effective coping strategies (Bahrololoum et al., 2012; Dalal, 2018; Law, 2004). The noted findings on female field-hockey players affirm the belief that emotional intelligent athletes can effectively regulate emotions to promote beneficial effects (Gross and Thompson, 2007) including coping under pressure (Laborde et al., 2011). It can, therefore, be argued that EI is a counterpart to adaptive qualities and a potential catapult for coping in senior level field-hockey in South Africa.

Notwithstanding the strong links noted between EI and coping ability in the present sample, it should be highlighted that national level field-hockey players revealed to be significantly better at managing their own emotions compared to their university level counterparts. This occurrence is not unusual as national/elite athletes have a proven tendency to apply better emotional regulation strategies than less successful/experienced athletes (Ruiz and Robazza, 2020). It is also recognised in the literature that professional athletes are fully aware of the detrimental effect unwanted emotions have on individual and overall team performance and consequently make it a priority to utilise effective coping strategies during competition (Davis et al., 2018). The fact that national team players were more likely to enjoy access to better support structures/services in a professional context with established social norms, defined players’ roles and responsibilities in comparison to university competitors could also contribute to better management of emotions (Tamminen et al., 2016). However, university level field hockey players revealed to be more coachable and utilise their emotions for problem solving better than national level players. They also exhibited better overall coping ability and dealings with adversity than their national level counterparts. As mentioned earlier, the level of coachability is often linked to the athlete’s motor skill level with less skilled athletes being more reliant on explicit coach instruction, support, and feedback compared to highly skilled athletes where sport-related learning and skills have reached a stage of implicit processing (Schmidt and Lee, 2019). Furthermore, the performance expectations, pressure, consequences of injury or failure, and training loads at university level hockey are foreseen to be significantly less to those experienced at a national level. Consequently, it is not improbable to find that university level players in the present sample were better at utilising their own emotions in solving problems. Reasoning for this could be based on research findings in football revealing that hours accumulated in practice negative correlated with players’ global scores of self-determination motivation (Hendry et al., 2019), thus, suggesting that national level players in our study could have felt less confident utilising their emotions in solving problems because of the increased demands and training loads they were exposed to. Related to the latter, Stewart and Meyers (2004) also point out that older elite level soccer players tended to be more motivated to avoid failure in comparison to younger players, which according to the Theory of Challenge and Threat States in Athletes (Meijen et al., 2020), could have severe implications for the players’ ability to cope with performance-related demands in sport.

Despite this study’s contribution to broader literature on EI and coping in female team sport athletes, several potential limitations need to be considered when interpreting our findings. First, the generalization of the results to participants of other team sports is limited due to the homogenous sample of the present study. Concluding remarks derived from a systematic review underscore that factors such as age, gender, ethnic origins, and cultural differences could have a large effect on the display and improvement of EI (Kotsou et al., 2019). Hence, future studies on EI in sport should examine its role within diverse athlete populations varying in age, ethnicity, and culture. Future research should also take players’ previous exposure to mental skills training into account as it could improve their coping ability in sport, which could inadvertently influence players’ awareness, understanding, and managing of emotions (EI). Furthermore, it should be kept in mind that the results are temporal to a specific time point in the competition season and might be different if longitudinally assessed across a period ranging from pre- to post-season. Evaluating athletes’ EI in relation to pressure levels experienced at different time points in a training season might offer practitioners added insight into the relevance of EI on coping ability in sport. Finally, the effect of EI interventions aimed at enhancing field-hockey players’ mental health and well-being in sport could further validate EI’s relevance to athletes’ coping ability. Regardless of these limitations, coaches and sport psychology consultants working in the context of high-level female field-hockey should consider incorporating aspects of EI in the pre-season screening as a supplementary prediction tool of players’ ability to deal with stressful demands in field-hockey.

## Conclusions

Our findings confirmed the predictive utility of EI concerning female field-hockey players’ coping ability in sport. The evidence from this study strengthens the view for potentially considering the use of EI testing and intervention in developing team athletes’ mental skills such as coping with adversity, coachability, concentration levels, overall confidence and achievement motivation. The aim of developing EI to improve players' coping ability may be an additional means to conventional mental skills training and considered a form of proactive behaviour, which may provide field-hockey players an added buffer against adversity, failure, and stress in their sport.

## Author Contributions

Conceptualization: N.T., A.K. and J.J.; methodology: N.T., A.K. and J.J.; software: J.J. and A.K.; validation: J.J. and A.K.; formal analysis: J.J. and A.K.; investigation: N.T.; resources: N.T. and A.K.; data curation: A.K. and N.T.; writing—original draft preparation: J.J., A.K. and N.T.; writing—review & editing: J.J., A.K. and N.T.; visualization: J.J. and A.K.; supervision: A.K. and J.J.; project administration: N.T. All authors have read and agreed to the published version of the manuscript.

## ORCID iD

Julius Jooste: 0000-0003-3278-0040

Ankebé Kruger: 0000-0002-4568-2346

Nicola Tinkler: 0000-0002-1682-0180
